# IDH1 mutation analysis – an example of putative glioma marker

**DOI:** 10.1186/1897-4287-10-S3-A14

**Published:** 2012-04-20

**Authors:** Marzena Lewandowska, T Szylberg, K Roszkowski, J Furtak, W Windorbska, J Rytlewska, W Jóźwicki

**Affiliations:** 1 Department of Tumor Pathology and Pathomorphology of the Franciszek Lukaszczyk Oncology Center, Bydgoszcz, Poland; 2 Department of Pathomorphology, Military Clinical Hospital, Bydgoszcz, Poland; 3 Department of Radiotherapy, The Franciszek Lukaszczyk Oncology Center, Bydgoszcz, Poland; 4 Department of Neurosurgery, Military Clinical Hospital, Bydgoszcz, Poland; 5 Department of Teleradiotherapy, The Franciszek Lukaszczyk Oncology Center, Bydgoszcz, Poland; 6 Department of Thoracic Surgery and Tumors, The Ludwik Rydygier Collegium Medicum, Nicolaus Copernicus University, Bydgoszcz, Poland; 7 Department of Tumor Pathology and Pathomorphology, The Ludwik Rydygier Collegium Medicum, Nicolaus Copernicus University, Bydgoszcz, Poland

## 

The astrocytoma cancer represents CNS neoplasms in which the predominant cell type is derived from an immortalized astrocyte. The genomewide analysis of glioma identified somatic mutation at codon 132 of the IDH1 gene which encodes NADP+ dependent isocitrate dehydrogenase. Further studies indicated that patients with somatic, heterozygous R132H mutation have distinct clinical characteristic: younger age at astrocytoma diagnosis (WHO II and WHO III) and improved clinical prognosis. Location of the majority of point mutations in the IDH1 gene are localized at 132 codon - what simplifies the use of this mutation for potential diagnostic purposes.

The presence of R132H IDH1 mutation was analysed in group of 38 patients diagnosed with: fibrillar astrocytoma, astrocytoma gemistocyticum, astrocytoma pilocyticum and astrocytoma anaplasticum. The IDH1 mutation status was determinated by immunohistochemistry using monoclonal antibody specific for the R132H mutation. Additional data verification was performed by HRM Cold-PCR and Sanger sequencing. For statistical evaluation we distinguished two subgroups of patients: with and without IDH1 R132H mutation. Presence of IDH1 mutation in Polish astrocytomas’ patients correlates with better clinical outcome and longer median overall survival. Our findings confirm overall tendency for better survival benefits in patients with IDH1 mutated tumors and indicates that presence or absence of IDH1 mutant proteins may become a potential target in personalized medicine.

